# Age-Related Differences in Resting-State EEG and Allocentric Spatial Working Memory Performance

**DOI:** 10.3389/fnagi.2021.704362

**Published:** 2021-11-04

**Authors:** Adeline Jabès, Giuliana Klencklen, Paolo Ruggeri, Jean-Philippe Antonietti, Pamela Banta Lavenex, Pierre Lavenex

**Affiliations:** ^1^Institute of Psychology, University of Lausanne, Lausanne, Switzerland; ^2^Faculty of Psychology, UniDistance Suisse, Brig, Switzerland

**Keywords:** electroencephalography, spontaneous brain activity, healthy aging, cognitive performance, spatial cognition

## Abstract

During normal aging resting-state brain activity changes and working memory performance declines as compared to young adulthood. Interestingly, previous studies reported that different electroencephalographic (EEG) measures of resting-state brain activity may correlate with working memory performance at different ages. Here, we recorded resting-state EEG activity and tested allocentric spatial working memory in healthy young (20–30 years) and older (65–75 years) adults. We adapted standard EEG methods to record brain activity in mobile participants in a non-shielded environment, in both eyes closed and eyes open conditions. Our study revealed some age-group differences in resting-state brain activity that were consistent with previous results obtained in different recording conditions. We confirmed that age-group differences in resting-state EEG activity depend on the recording conditions and the specific parameters considered. Nevertheless, lower theta-band and alpha-band frequencies and absolute powers, and higher beta-band and gamma-band relative powers were overall observed in healthy older adults, as compared to healthy young adults. In addition, using principal component and regression analyses, we found that the first extracted EEG component, which represented mainly theta, alpha and beta powers, correlated with spatial working memory performance in older adults, but not in young adults. These findings are consistent with the theory that the neurobiological bases of working memory performance may differ between young and older adults. However, individual measures of resting-state EEG activity could not be used as reliable biomarkers to predict individual allocentric spatial working memory performance in young or older adults.

## Introduction

Normal aging, in absence of evidence of mild cognitive impairment (MCI) or dementia, has been associated with an overall decline in working memory performance (Fabiani, 2012). Working memory refers to processes that enable temporary storage and manipulation of information necessary for cognitive processes such as language comprehension, learning and reasoning (Baddeley, 1992), and which are resistant to interference and distraction during the retention interval (Bizon et al., 2012; Banta Lavenex et al., 2014; Spellman et al., 2015). Age-related changes in working memory performance affect verbal, visual and spatial information (Shelton et al., 1982; Salthouse, 1995; Myerson et al., 1999; Jenkins et al., 2000; Chen et al., 2003). It is also well established that spontaneous brain activity at rest changes across the lifespan, and it has been suggested that such changes may reflect age-related cognitive declines (see Anderson and Perone, 2018 for a recent review). However, the exact nature of the alterations in resting-state brain dynamics that have been reported in the literature is unclear. Indeed, different electroencephalography (EEG) recording and analysis methods have been used, and different EEG parameters have been reported. In addition, different recording conditions (i.e., eyes closed or eyes open) and different age ranges have been considered (Supplementary Materials 1–4). Consequently, an experimental re-evaluation of differences in resting-state EEG activity between young and older adults, in which numerous complementary parameters are used to analyze all four major frequency bands in both the eyes open and eyes closed conditions is warranted. Such an analysis may help to provide a coherent framework in which to consider previous results and clarify whether some parameter(s) of resting-state brain dynamics may be associated with age-related differences in spatial working memory performance.

### Resting-State EEG Activity and Working Memory

Previous studies evaluating how resting-state EEG parameters correlate with working memory performance have produced varied results, depending on the EEG parameters and the working memory measures considered (Table 1). Moreover, although a number of correlations were reported between some EEG parameters and some measures of memory performance, these correlations were usually insufficient to make the types of individual predictions that are necessary for use in clinical settings.

**TABLE 1:** Studies reporting findings on the possible links between age-related changes in resting-state frequency bands activity and working memory performance: positive (Pos), negative (Neg) or no link (None).

**Study**	**Age**	**Tasks used**	**Method**		**Theta**	**Alpha**	**Beta**	**Gamma**
**Young adults**								
Oswald et al., 2017	26 ± 5	Letter-number sequencing	MEG eyes open	Abs. power	**Pos**, 4–8 Hz	**Pos**, 8–13 Hz	**Pos**, 13–30 Hz	**Pos**, >60 Hz
		Digit span			None, 4–8 Hz	**Pos**, 8–13 Hz	**Pos**, 13–30 Hz	**Pos**, >60 Hz
		Spatial addition			**Pos**, 4–8 Hz	**Pos**, 8–13 Hz	**Pos**, 13–30 Hz	**Pos**, 30–60, >60 Hz
Vlahou et al., 2014	18–54	Trail Making test A	MEG eyes open	Abs. power	None, 4.5–7.5 Hz	None, IAPF		
		Trail Making test B			None, 4.5–7.5 Hz	None, IAPF		
Heister et al., 2013	22 ± 3	Verbal n-back	MEG eyes open and closed	Abs. power	None, 1–7 Hz (delta/theta)	None, 8–13 Hz	None, 14–30 Hz	
Trammell et al., 2017	21 ± 1	Words list recall	EEG eyes closed	Rel. power	None, 4–8 Hz	None, IAPF + 8–12 Hz	None, 12–24 Hz	
**Older adults**								
Trammell et al., 2017	73 ± 3	Words list recall	EEG eyes closed	Rel. power	None, 4–8 Hz	None, IAPF + 8–12 Hz	None, 12–24H z	
Vlahou et al., 2014	55–89	Trail Making test A	MEG eyes open	Abs. power	None, 4.5–7.5 Hz	None, IAPF		
		Trail Making test B			**Pos**, 4.5–7.5 Hz	None, IAPF		
Roca-Stappung et al., 2012	67 ± 7	Working memory index (WAIS-III)	EEG eyes closed	Abs. power Rel. power	**Neg**, 4–7.5 Hz None, 4–7.5Hz	None, 8–12.5 Hz **Pos**, 8–12.5 Hz	None, 13–19 Hz None, 13–19 Hz	
Finnigan and Robertson, 2011	61 (56–70)	Digit span forward	EEG eyes closed	Rel. power	None, 4–6.5 Hz	None, 7.5–12 Hz + IAPF	None, 13–30 Hz	
		Digit span backward			None, 4–6.5 Hz	None, 7.5–12 Hz + IAPF	**Neg,** 13–30 Hz at PZ	
van der Hiele et al., 2008	70 ± 5	Trail making test A	EEG eyes closed	Rel. power	**Neg,** 4–8Hz			
		WMS			None			
**Throughout adulthood**								
Reichert et al., 2016	25 vs. 48 vs. 68	City map recall	EEG eyes closed and open	Rel. power	None, 4–8 Hz	None, 8–10 HZ **Pos,** 10–12Hz	None, 12–35 Hz	None, 35–45 Hz
Richard Clark et al., 2004	11–70	Digit span forward	EEG eyes closed	Abs. power		None, 8–13 Hz + IAPF		
		Reversed digit span				None, 8–13Hz + IAPF		

For example, in young adults, Oswald et al. (2017) reported that spontaneous MEG theta, alpha, beta and gamma power, recorded in the eyes open condition, correlated positively with some subtests of the Wechsler Adult Intelligence Scale (WAIS-IV) and the Wechsler Memory Scale (WMS-IV; Wechsler, 2008) designed to assess working memory performance. In contrast, other studies have failed to find correlations between spontaneous resting-state brain activity and working memory performance in healthy young adults (Heister et al., 2013; Vlahou et al., 2014; Trammell et al., 2017).

In older adults, various results have also been reported (Table 1). Trammell et al. (2017) found no correlations between eyes-closed resting-state EEG individual alpha peak frequency, theta, alpha and beta relative power and word list immediate recall. Vlahou et al. (2014) found a positive correlation between eyes-open resting-state MEG theta power and performance on the Trail Making Test B, which is thought to assess primarily working memory ability (Sanchez-Cubillo et al., 2009). In contrast, Roca-Stappung et al. (2012) found a negative correlation between eyes-closed EEG theta absolute power (but not relative power) and working memory index (WMI) score (Wechsler, 1997), a positive correlation between alpha relative power (but not absolute power) and WMI score, and no correlation between beta absolute or relative power and WMI score. van der Hiele et al. (2008) found a negative correlation between eyes-closed EEG theta relative power and performance on the Trail Making Test A, and no correlation between eyes-closed EEG theta relative power and the Wechsler Memory Scale (WMS). These results further contrast with those of Finnigan and Robertson (2011), who reported no correlation between eyes-closed EEG theta relative power and forward/backward digit span performance, and a negative correlation between beta relative power measured at Pz and backward digit span scores.

In sum, although some working memory or related cognitive capacities have been correlated to some extent with theta-, alpha-, beta- and gamma-band activity, no clear hypothesis specifying which resting-state frequency band activity may actually predict *individual* working memory performance can be formulated from the results of previous studies. Moreover, although some data suggest that some correlations between resting-state brain activity and working memory performance may differ between young and older adults (Vlahou et al., 2014), clear predictions about these differences cannot be formulated at this point.

### Aging and Spatial Working Memory

Despite the wide variety of memory tasks reported in the literature, to our knowledge only one study in humans considered the possible links between resting-state EEG activity and the types of visuo-spatial working memory that may underlie navigational strategies. Reichert et al. (2016) compared 21 young individuals (22–33 years), 25 middle-aged individuals (40–59 years) and 24 older individuals (60–83 years). EEG was recorded at rest, prior to memorizing a route between two buildings presented on a map. Subjects were then asked to recall the route by drawing it on an empty map right after encoding. Across all age groups, higher alpha II (10–12 Hz) relative power recorded with the eyes closed was associated with better immediate route recall, whereas alpha I (8–10 Hz), beta (12–35 Hz), theta (4–8 Hz), or gamma (35–45 Hz) activity did not correlate with performance. However, these authors did not test the correlations between alpha II relative power and working memory performance in each age group separately (see also Richard Clark et al., 2004 for similar results across ages). Indeed, as was the case for some of the studies described above, because both resting-state brain activity and memory performance vary with age, such correlations may have been driven by differences between age groups and therefore do not necessarily reveal a direct link between brain activity in particular frequency bands and memory performance.

Moreover, Reichert et al.’s task, presented on a paper map, assessed egocentric spatial representations, which involve distinct brain networks from allocentric spatial representations such as those built when locomoting in a real-world environment. Indeed, the brain can represent locations via distinct spatial representation systems (O’keefe and Nadel, 1978; Burgess, 2006; Banta Lavenex and Lavenex, 2009). Allocentric spatial memory is the ability to encode and recall one’s own position, as well as the position of other objects and locations, relative to distal objects and locations in the environment, and provides the foundation for the formation of cognitive maps (O’keefe and Nadel, 1978; Burgess, 2006; Banta Lavenex and Lavenex, 2009). Accordingly, we previously compared the performance of 20–30-year-old and 65–75-year-old healthy adults who had to learn the locations or colors of three pads among 18 pads distributed on the floor in a real-world laboratory environment, and found that older adults performed less well than young adults on both allocentric spatial and color working memory tasks (Klencklen et al., 2017a,b). We proposed that age-related differences in working memory performance may be most influenced by memory load, as well as the representational demands of the task and its dependence on hippocampal function, rather than by the type of information to be remembered (i.e., spatial vs. color information). These results thus raised questions about the functional brain networks that may contribute to age-related differences in spatial working memory performance.

Since aging may differentially impact different memory systems or circuits (Gallagher et al., 2006; Tomas Pereira et al., 2015; Ash et al., 2016; Zhong and Moffat, 2018), it is important to expand the investigation of the potential relationships between age-related differences in resting-state brain activity and working memory performance to tasks that implicate the hippocampal formation, such as allocentric spatial working memory tasks (Banta Lavenex et al., 2015). Moreover, since allocentric spatial working memory tasks have been commonly used in studies with rodents (O’keefe and Nadel, 1978; Rapp and Gallagher, 1996; Morris, 2007; Ash et al., 2016), including their use in humans can serve as a critical bridge to compare the results from these two complementary domains of research.

### Aims of the Study

We recorded resting-state brain activity and tested allocentric spatial working memory in healthy young (20–30 years) and older (65–75 years) adults. We adapted standard EEG methods to record brain activity in mobile participants in a non-shielded environment, in both eyes closed and eyes open conditions.

The first aim of the study was to provide a re-evaluation of some age-related differences in resting-state brain activity that may help to clarify some presumed inconsistencies found in the literature, which may be linked to the recording conditions (i.e., eyes closed vs. eyes open) and the specific parameters that were reported. We extracted and analyzed resting-state EEG parameters most-commonly reported in the literature (i.e., peak frequency, absolute and relative power) for the theta, alpha, beta and gamma frequency bands.

The second aim of the study was to determine whether resting-state brain activity can reliably predict *individual* working memory performance in young and older adults. Based on some previous findings described above, we hypothesized that different EEG measures of resting-state brain activity may correlate with allocentric spatial working memory performance in young and older individuals.

## Materials and Methods

### Participants

**FIGURE 1:**
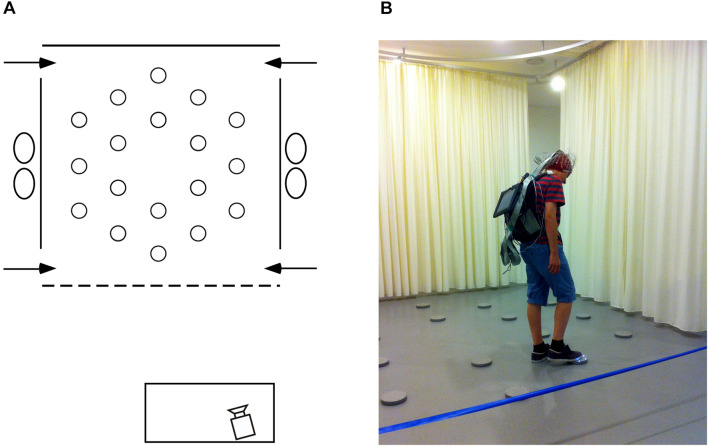
Testing environment. **(A)** Schematic, aerial view of the arena (3.64 × 3.64 m) within the experimental room (8 × 8 m). At each of the four near and far corners of the curtained arena a 50 cm gap served as one of the four different entry points (arrows) through which participants must pass in order to enter and exit the testing arena. Eighteen foot pads were regularly arranged on the floor of the arena. **(B)** Picture of the arena with a participant carrying the EEG recording system and touching an illuminating foot pad during the allocentric spatial working memory task.

Twenty-one young adults (12 females) aged 20–30 years (M: 26.32, SD: 3.48) and 27 older adults (10 females) aged 65–75 years (M: 71.59, SD: 3.36) were recruited, via personal connections, email posting on social networks and via flyers distributed through local senior organizations. Care was taken to recruit participants from all education levels. Exclusion criteria were subjective memory complaints (Schmand et al., 1996; Jonker et al., 2000), and a history of learning disabilities, visual perception disabilities, left-handedness, birth complications, neurological medication, a history of neurological or psychiatric disease, and trauma. All participants (but one young and two older adults) participated in previous studies (Klencklen et al., 2017a,b) and were screened at the time (2 years prior to the current experiment) for dementia by a neuropsychologist (G.K.), using a battery of neuropsychological tests including: general cognitive status with the Mini Mental State Examination (MMSE; Folstein et al., 1975); the Progressive Matrice-12 (Raven et al., 2003); the Vocabulary, Digit Spans, Arithmetic and Similitude sub-tests from the Wechsler Adult Intelligence Scale-III (WAIS-III; Wechsler, 1997); color vision with the Ishihara test (Ishihara, 1917); and the Corsi Block-Tapping test Forward and Backward (Corsi, 1972). For each test, older participants were found to be within 1.75 standard deviations of the norm for age-matched controls (Klencklen et al., 2017a,b). At the time of the present study, normal cognitive capacities were inferred using participants’ self-report, as well as by comparing the spatial working memory performance of older individuals who were tested in both the present study and the study 2 years prior (Klencklen et al., 2017a; paired *t*-tests: CBE: *t*_(22)_ = 1.664, *p* = 0.110, *d*_*z*_ = 0.355; NET: *t*_(22)_ = 1.811, *p* = 0.084, *d*_*z*_ = 0.386). We also compared the results of all the older participants tested in the current study with those of all the older participants tested previously (Klencklen et al., 2017a; unpaired *t*-tests. CBE: *t*_(57)_ = 0.508, *p* = 0.613, *d*_*s*_ = 0.135; NET: *t*_(57)_ = 0.757, *p* = 0.452, *d*_*s*_ = 0.201). All participants were tested for about 2 h between 8 A.M. and 8 P.M. They gave written informed consent prior to beginning the study and were compensated monetarily for their participation. Human subjects research was approved by the Cantonal Ethics Committee (Vaud, Switzerland, Protocol No 384/15). The individual in Figure 1 of this manuscript has given written informed consent to publish this photograph.

### Spatial Working Memory Task

Participants were tested at the University of Lausanne in a large square room (8 m × 8 m; Figure 1A) containing many polarizing features such as a door, a table, chairs and folding room-dividing screens. Detailed description of the testing facility and procedure has been published previously (Klencklen et al., 2017a,b). Briefly, within the room a 3.64 m × 3.64 m testing arena contained 18 visually identical, circular gray foot pads equipped with L.E.D. lights. Foot pads designated as goals by the experimenters would illuminate when touched lightly with the foot, but the light would extinguish as soon as the foot was removed from the pad. All testing was videotaped with a video camera located in front of the testing arena. Participants were given 10 trials during which they had to learn three predetermined goal locations on each trial. Each trial consisted of two phases: during the first encoding phase, participants had to explore the arena to discover the three goal locations, touching each disk in order to identify, learn and remember the locations of the illuminating disks. During a 90-s inter-phase interval, participants were required to mentally count backward by one from a predetermined number. After the inter-phase interval, the recall phase began. Participants were asked to indicate the three goal locations, in no particular order, by walking to each disk and stepping on it to illuminate it. The same procedure was repeated for 10 trials with three new and non-adjacent goal locations predetermined pseudo-randomly for each trial.

### Resting-State EEG Recordings and Analyses

#### Recordings

A 128-channel Biosemi Active Two system (Biosemi, Amsterdam, Netherlands) was used to record spontaneous resting-state brain activity just minutes before the spatial working memory task began. Surgical caps according to the Biosemi designed equiradial electrode placement system (“ABC” layout) were used. A backpack was designed to carry the recording system, allowing participants to freely move and perform the task immediately after recording (Figure 1B), as well as to record brain electrical activity during the inter-phase intervals (memory maintenance) which will be the focus of a subsequent report. A Sony tablet running the ActiView software (version 7.05) was fixed to the front side of the backpack, allowing constant monitoring of the EEG recording. For the resting-state recordings described in this manuscript, prior to starting the memory task, participants were seated on a stool on the left side of the arena where ambient electrical noise was minimal. Participants were instructed to relax and move as little as possible. Six 1-min baseline recordings, alternating eyes-closed and eyes-open for 1 min each, were performed at a sampling rate of 2,048 Hz. The level of DC offset was checked (±20 mV) before data collection. Right, left, and middle-orbital flat electrodes were used to monitor eye movements and blinks. Raw EEG data were down-sampled offline to 512 Hz using the Biosemi decimator tool for further analysis. All EEG recording data are freely available at: 10.5281/zenodo.3875159.

#### EEG Frequency Bands Analysis

Resting-state power analysis was performed with BrainVision Analyzer software 2.1 (Brain Products, Munich, Germany). A 1 Hz high-pass filter (IIR Filter Butterworth, order 2) and a 50 Hz Notch filter were first applied. Data were then visually inspected and segments containing residual artifacts (except blinks and eye movement artifacts) were manually removed. Independent Component Analysis (ICA) was performed to correct eye movements, blinks and cardiac artifacts. Topographic interpolation (3D spherical spline) was used to correct channels that were noisy throughout the recording (max. 10). Data were then average-referenced, and segmented into 1 s epochs. A semi-automatic artifact rejection tool was used to identify residual artifacts. Artifact-free EEG segments were then analyzed with a fast Fourier transform (FFT), using a Hanning window (window length 10%) to compute the power spectra (μV^2^/Hz) of the eyes-open and eyes-closed conditions (0.5 Hz frequency resolution). Power spectra were computed for four frequency bands: alpha, theta, beta and gamma. Because alpha frequency varies as a function of age, we defined the alpha-band individually for each participant, according to Klimesch’s description (Klimesch, 1999; see also Scally et al., 2018 for a discussion on this topic). First, we identified the individual dominant alpha peak frequency for each participant in each eye condition. Individual alpha-bands were defined, with a width of 3 Hz below the peak and 2 Hz above the peak. The theta-band was defined as a 3 Hz band starting at the lower edge of the alpha-band. Beta- and gamma-bands were defined as fixed bands according to the literature (14–28 Hz and 30–47 Hz, respectively). Three older participants did not exhibit a clear alpha peak (6% of total sample). It has been previously reported that individuals with normal brain function might not show a typical alpha peak (2.7% in Chiang et al., 2011). As we could not define alpha- and theta-bands using this method for these three participants, and as our conclusions for beta and gamma activity did not change by removing these three participants, we did not include them in the final sample.

### Statistical Analyses

Statistical analyses were performed using IBM SPSS Statistics for Mac, version 25 (IBM Corp., Armonk, NY, United States; General Linear Model (GLM) analyses with repeated-measures and *t*-tests) and R software version 3.5.2 (R Core Team, 2018; Principal Component Analysis and multiple regression analyses). We did not use corrections for multiple comparisons for individual tests (see below) because in our study the risk of reporting a difference that may not exist (type I error) is not worse than the risk of missing a difference that may exist (type II error). Accordingly, we followed the recommendations of Rothman (1990), who argued that *“not making adjustments for multiple comparisons is preferable because it will lead to fewer errors of interpretation when the data under evaluation are not random numbers but actual observations on nature*,” and Saville (1990), who also argued that a procedure without correction is preferable because it provides greater consistency to compare results between studies. For ANOVAs, we report effect size with η*^2^_*p*_* (partial eta squared, as reported by SPSS 25.0). We report effect size with Cohen’s d_*s*_ for independent samples *t*-tests, and Cohen’s d_*z*_ for paired samples *t*-tests (Lakens, 2013).

#### Resting-State EEG Frequency Bands

For each frequency band, mean absolute power and relative power (absolute power of the band divided by the total power from 2 to 47 Hz), peak frequency and power (alpha), averaged peak frequency and power (theta and beta), as well as the mean total power (2–47 Hz) were calculated for each individual in both eye conditions. Averaged peak frequency (or center of gravity) was defined as: [Sum (a(f) × f))/(Sum a(f)], where a(f) is the power estimate at frequency f (Klimesch et al., 1993). The averaged peak power was the power value at the averaged peak frequency. EEG parameters were analyzed using a GLM analysis with a between-subjects factor (two groups: young, older) and repeated measures (two eye conditions: closed, open). Independent samples *t*-tests were used to compare EEG parameters between young and older adults in each eye condition. As we found only marginal effects of sex and no interactions between eye conditions, age groups and sex for any parameters, data from men and women were combined for presentation.

#### Allocentric Spatial Working Memory

The following measures were used (Banta Lavenex et al., 2014): (1) the number of goal disks visited before making an error (i.e., visiting a non-goal disk, CBE: correct before error), an estimate of memory capacity; and (2) the number of errorless trials (NET), an estimate of perfect memory. We used independent samples *t*-tests to compare performance between young and older adults.

#### Resting-State EEG and Working Memory

In order to test whether different resting-state EEG signatures may correlate with allocentric spatial working memory performance in young or older adults, we performed a Principal Component Analysis (PCA, no rotation; Abdi and Williams, 2010) on all 30 EEG variables (Supplementary Material 5) from all participants. Horn’s Parallel Analysis for component retention (900 iterations, 95 centile estimate) revealed four components with an adjusted Eigenvalue >1. These four components explained 80.52% of the total variance and were retained for further analysis. We then performed multiple regression analyses with the four EEG components extracted from the PCA and the two measures of memory performance (CBE and NET). These 4-component models were then simplified using a step by step minimization of Akaike Information Criterion (AIC, Sakamoto et al., 1986). These parsimonious models described the data as well as the 4-component models (CBE: Δ *R*^2^ = 0.02, *F*_(3__,35)_ = 0.581, *p* = 0.631; NET: Δ *R*^2^ = 0.02, *F*_(2__,35)_ = 0.612, *p* = 0.548). Finally, based on the significant interactions between age groups and individual components of the PCA, we further determined whether the regression slope differed from zero for each age group.

## Results

### Resting-State EEG

#### Theta-Band

**FIGURE 2:**
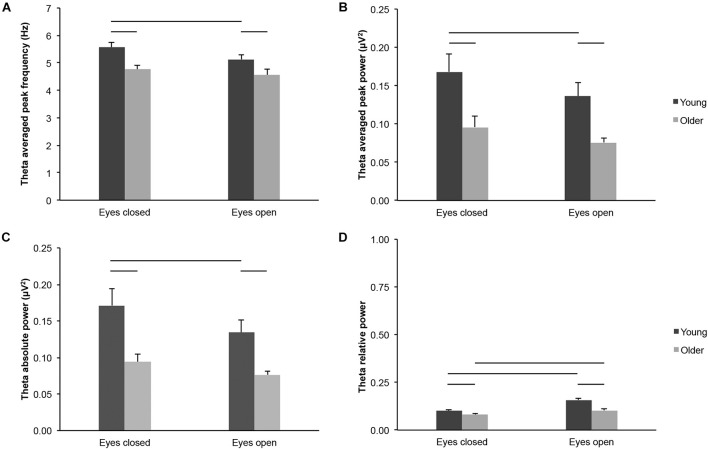
Theta-band activity measured across the scalp in healthy 20–30-year-old (Young; dark gray bars) and 65–75-year-old (Older; light gray bars) adults. **(A)** Theta averaged peak frequency. **(B)** Theta averaged peak power. **(C)** Theta absolute power. **(D)** Theta relative power. Average ± SEM. Horizontal bars indicate statistically significant differences between age groups or eye conditions (*p* < 0.05).

We found lower theta-band frequency and power measured across the scalp in 65–75-year-old adults as compared to 20–30-year-old adults, in both eyes open and eyes closed conditions: Theta averaged peak frequency (Figure 2A; age groups: *F*_(1,43)_ = 9.047, *p* = 0.004, young > older, η*^2^_*p*_* = 0.174; age groups × eye conditions: *F*_(1,43)_ = 0.921, *p* = 0.343, η*^2^_*p*_* = 0.021; eyes closed: young > older, *t*_(43)_ = 3.248, *p* = 0.002, *d*_*s*_ = 0.971; eyes open: young > older, *t*_(43)_ = 2.091, *p* = 0.042, *d*_*s*_ = 0.625), theta averaged peak power (Figure 2B; age groups: *F*_(1,43)_ = 10.537, *p* = 0.002, young > older, η*^2^_*p*_* = 0.197; age groups x eye conditions: *F*_(1,43)_ = 0.437, *p* = 0.512, η*^2^_*p*_* = 0.010; eyes closed: young > older, *t*_(43)_ = 2.718, *p* = 0.009, *d*_*s*_ = 0.812; eyes open: young > older, *t*_(43)_ = 3.563, *p* = 0.001, *d*_*s*_ = 1.065), and theta absolute power (Figure 2C; age groups: *F*_(1,43)_ = 11.805, *p* = 0.001, young > older, η*^2^_*p*_* = 0.215; age groups × eye conditions: *F*_(1,43)_ = 1.852, *p* = 0.181, η*^2^_*p*_* = 0.041; eyes closed: young > older, *t*_(43)_ = 3.130, *p* = 0.003, *d*_*s*_ = 0.935; eyes open: young > older, *t*_(43)_ = 3.563, *p* = 0.001, *d*_*s*_ = 1.065). Theta relative power was also lower in older adults than in young adults (Figure 2D; age groups: *F*_(1,43)_ = 22.635, *p* < 0.001, young > older, η*^2^_*p*_* = 0.169), and this difference appeared more important in the eyes open condition (age groups × eye conditions: *F*_(1,43)_ = 8.722, *p* = 0.005, η*^2^_*p*_* = 0.169; eyes closed: young > older, *t*_(__43__)_ = 2.310, *p* = 0.026, *d*_*s*_ = 0.690; eyes open: young > older, *t*_(__43__)_ = 5.029, *p* < 0.001, *d*_*s*_ = 1.503).

Several parameters reflected different theta-band activity in the eyes open and eyes closed conditions across age groups, but not necessarily for each group considered separately: Theta averaged peak frequency (Figure 2A; eye conditions: *F*_(1,43)_ = 6.665, *p* = 0.013, open < closed, η*^2^_*p*_* = 0.134; young: open < closed, *t*_(20)_ = 2.821, *p* = 0.011, *d*_*z*_ = 0.616; older: open < closed, *t*_(23)_ = 1.072, *p* = 0.295, *d*_*z*_ = 0.219), theta averaged peak power (Figure 2B; eye conditions: *F*_(1,43)_ = 8.642, *p* = 0.005, open < closed, η*^2^_*p*_* = 0.167; young: open < closed, *t*_(20)_ = 2.733, *p* = 0.013, *d*_*z*_ = 0.596; older: open < closed, *t*_(23)_ = 1.547, *p* = 0.135, *d*_*z*_ = 0.316), and theta absolute power (Figure 2C; eye conditions: *F*_(1,43)_ = 14.899, *p* < 0.001, open < closed, η*^2^_*p*_* = 0.257; young: open < closed, *t*_(20)_ = 3.201, *p* = 0.004, *d*_*z*_ = 0.699; older: open < closed, *t*_(23)_ = 2.064, *p* = 0.050, *d*_*z*_ = 0.421). Theta relative power was higher in the eyes open condition than in the eyes closed condition for both young and older adults (Figure 2D; eye conditions: *F*_(1,43)_ = 44.189, *p* < 0.001, open > closed, η*^2^_*p*_* = 0.507; young: open > closed, *t*_(20)_ = 4.949, *p* < 0.001, *d*_*z*_ = 1.080; older: open > closed, *t*_(23)_ = 4.669, *p* < 0.001, *d*_*z*_ = 0.953).

#### Alpha-Band

**FIGURE 3:**
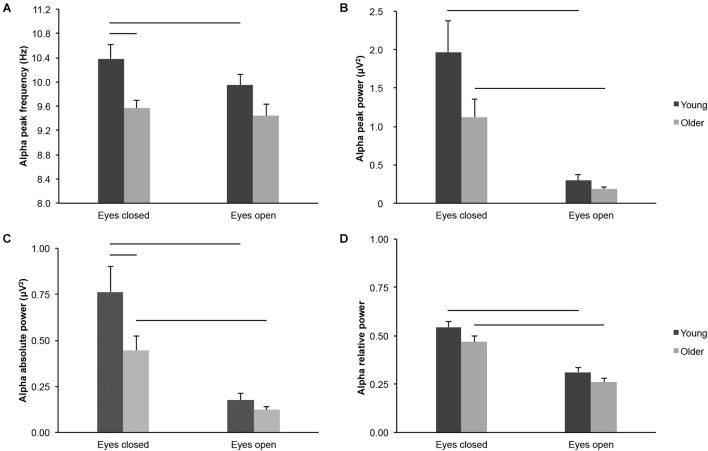
Alpha-band activity measured across the scalp in healthy 20–30-year-old (Young; dark gray bars) and 65–75-year-old (Older; light gray bars) adults. **(A)** Alpha peak frequency. **(B)** Alpha peak power. **(C)** Alpha absolute power. **(D)** Alpha relative power. Average ± SEM. Horizontal bars indicate statistically significant differences between age groups or eye conditions (*p* < 0.05).

Several parameters reflected differences in alpha-band activity across the scalp in older adults as compared to young adults, across eye conditions, but not necessarily in each condition considered separately: Alpha peak frequency (Figure 3A; age groups: *F*_(1,43)_ = 8.463, *p* = 0.006, young > older, η*^2^_*p*_* = 0.164; age groups × eye conditions: *F*_(1,43)_ = 1.334, *p* = 0.254, η*^2^_*p*_* = 0.030; eyes closed: young > older, *t*_(43)_ = 3.170, *p* = 0.003, *d*_*s*_ = 0.947; eyes open: young > older, *t*_(43)_ = 1.907, *p* = 0.063, *d*_*s*_ = 0.570), alpha peak power (Figure 3B; age groups: *F*_(1,43)_ = 3.908, *p* = 0.054, young > older, η*^2^_*p*_* = 0.083; age groups x eye conditions: *F*_(1,43)_ = 2.596, *p* = 0.114, η*^2^_*p*_* = 0.057; eyes closed: young > older, *t*_(43)_ = 1.828, *p* = 0.075, *d*_*s*_ = 0.546; eyes open: young > older, *t*_(43)_ = 1.337, *p* = 0.188, *d*_*s*_ = 0.400), alpha absolute power (Figure 3C; age groups: *F*_(1,43)_ = 4.536, *p* = 0.039, young > older, η*^2^_*p*_* = 0.095; age groups x eye conditions: *F*_(1,43)_ = 3.321, *p* = 0.075, η*^2^_*p*_* = 0.072; eyes closed: young > older, *t*_(43)_ = 2.047, *p* = 0.047, *d*_*s*_ = 0.612; eyes open: young > older, *t*_(43)_ = 1.352, *p* = 0.183, *d*_*s*_ = 0.404), and alpha relative power (Figure 3D; age groups: *F*_(_*_1,43_*_)_ = 3.931, *p* = 0.054, young > older, η*^2^_*p*_* = 0.084; age groups × eye conditions: *F*_(1,43)_ = 0.309, *p* = 0.581, η*^2^_*p*_* = 0.007; eyes closed: young > older, *t*_(43)_ = 1.748, *p* = 0.088, *d*_*s*_ = 0.522; eyes open: young > older, *t*_(43)_ = 1.599, *p* = 0.117, *d*_*s*_ = 0.478).

Several parameters of alpha-band activity were lower in the eyes open condition than in the eyes closed condition across age groups, but not necessarily for each group considered separately: Alpha peak frequency (Figure 3A; eye conditions: *F*_(1,43)_ = 4.436, *p* = 0.041, open < closed, η*^2^_*p*_* = 0.094; young: open < closed, *t*_(20)_ = 2.295, *p* = 0.033, *d*_*z*_ = 0.501; older: open < closed, *t*_(23)_ = 0.681, *p* = 0.503, *d*_*z*_ = 0.139), alpha peak power (Figure 3B; eye conditions: *F*_(1,43)_ = 33.027, *p* < 0.001, open < closed, η*^2^_*p*_* = 0.434; young: open < closed, *t*_(20)_ = 3.998, *p* = 0.001, *d*_*z*_ = 0.872; older: open < closed, *t*_(23)_ = 4.328, *p* < 0.001, *d*_*z*_ = 0.883), alpha absolute power (Figure 3C; eye conditions: *F*_(1,43)_ = 39.759, *p* < 0.001, open < closed, η*^2^_*p*_* = 0.480; young: open < closed, *t*_(20)_ = 4.390, *p* < 0.001, *d*_*z*_ = 0.958; older: open < closed, *t*_(23)_ = 4.776, *p* < 0.001, *d*_*z*_ = 0.975), and alpha relative power (Figure 3D; eye conditions: *F*_(1,43)_ = 111.357, *p* < 0.001, open < closed, η*^2^_*p*_* = 0.721; young: open < closed, *t*_(20)_ = 6.840, *p* < 0.001, *d*_*z*_ = 1.493; older: open < closed, *t*_(23)_ = 8.209, *p* < 0.001, *d*_*z*_ = 1.676).

#### Beta-Band

**FIGURE 4:**
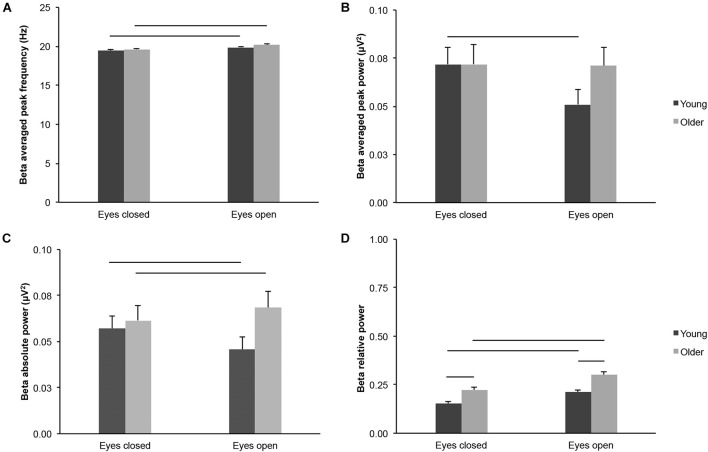
Beta-band activity measured across the scalp in healthy 20–30-year-old (Young; dark gray bars) and 65–75-year-old (Older; light gray bars) adults. **(A)** Beta averaged peak frequency. **(B)** Beta averaged peak power. **(C)** Beta absolute power. **(D)** Beta relative power. Average ± SEM. Horizontal bars indicate statistically significant differences between age groups or eye conditions (*p* < 0.05).

We did not find consistent age-group differences for most absolute measures of beta-band activity across the scalp. Beta averaged peak frequency did not differ between young and older adults irrespective of eye condition (Figure 4A; age groups: *F*_(1,43)_ = 2.142, *p* = 0.151, η*^2^_*p*_* = 0.047; age groups x eye conditions: *F*_(1,43)_ = 0.625, *p* = 0.434, η*^2^_*p*_* = 0.014; eyes open: *t*_(43)_ = 1.596, *p* = 0.118, *d*_*s*_ = 0.477; eyes closed: *t*_(43)_ = 1.176, *p* = 0.246, *d*_*s*_ = 0.351). Beta averaged peak power did not differ between young and older adults irrespective of eye condition (Figure 4B; age groups: *F*_(1,43)_ = 0.619, *p* = 0.436, η*^2^_*p*_* = 0.014; eyes open: *t*_(43)_ = 1.617, *p* = 0.113, *d*_*s*_ = 0.483; eyes closed: *t*_(43)_ = 0.002, *p* = 0.998, *d*_*s*_ = 0.001), but there was an interaction between age groups and eye conditions (*F*_(1,43)_ = 8.075, *p* = 0.007, η*^2^_*p*_* = 0.158; see below). Beta absolute power did not differ between young and older adults irrespective of eye condition (Figure 4C; age groups: *F*_(1,43)_ = 1.624, *p* = 0.209, η*^2^_*p*_* = 0.036), but there was an interaction between age groups and eye conditions (*F*_(1,43)_ = 10.534, *p* = 0.002, η*^2^_*p*_* = 0.197). There was no difference between age groups in the eyes closed condition (*t*_(43)_ = 0.427, *p* = 0.671, *d*_*s*_ = 0.128), but the difference was nearly significant in the eyes open condition (young < older, *t*_(43)_ = 1.968, *p* = 0.056, *d*_*s*_ = 0.588). Beta relative power was higher in older adults than in young adults in both eye conditions (Figure 4D; age groups: *F*_(1,43)_ = 19.559, *p* < 0.001, young < older, η*^2^_*p*_* = 0.313; age groups × eye conditions: *F*_(1,43)_ = 2.130, *p* = 0.152, η*^2^_*p*_* = 0.047; eyes open: young < older, *t*_(43)_ = 4.614, *p* < 0.001, *d*_*s*_ = 1.379; eyes closed: young < older, *t*_(43)_ = 3.545, *p* = 0.001, *d*_*s*_ = 1.059).

Beta averaged peak frequency was higher in the eyes open condition than in the eyes closed condition for both age groups (Figure 4A; eye conditions: *F*_(1,43)_ = 84.870, *p* < 0.001, open > closed, η*^2^_*p*_* = 0.664; young: open > closed, *t*_(20)_ = 5.434, *p* < 0.001, *d*_*z*_ = 1.186; older: open > closed, *t*_(23)_ = 7.759, *p* < 0.001, *d*_*z*_ = 1.584). In contrast, beta averaged peak power was lower in the eyes open condition than in the eyes closed condition for young adults, but not for older adults (Figure 4B; eye conditions: *F*_(1,43)_ = 9.552, *p* = 0.003, open < closed, η*^2^_*p*_* = 0.182; see interaction above; young, open < closed, *t*_(20)_ = 3.404, *p* = 0.003, *d*_*z*_ = 0.743; older, open < closed, *t*_(23)_ = 0.229, *p* = 0.821, *d*_*z*_ = 0.047). Beta absolute power did not differ between eye conditions across age groups (Figure 4C; eye conditions: *F*_(1,43)_ = 0.613, *p* = 0.438, η*^2^_*p*_* = 0.014). However, there was an interaction between age groups and eye conditions (see above): beta absolute power was lower in the eyes open condition for young adults (open < closed, *t*_(20)_ = 2.418, *p* = 0.025, *d*_*z*_ = 0.528), whereas beta absolute power was higher in the eyes open condition for older adults (open > closed, *t*_(23)_ = 2.098, *p* = 0.047, *d*_*z*_ = 0.428). Finally, beta relative power was higher in the eyes open condition than in the eyes closed condition, for both age groups (Figure 4D; eye conditions: *F*_(1,43)_ = 80.342, *p* < 0.001, open > closed, η*^2^_*p*_* = 0.651; young: open > closed, *t*_(20)_ = 6.285, *p* < 0.001, *d*_*z*_ = 1.371; older: open > closed, *t*_(23)_ = 6.721, *p* < 0.001, *d*_*z*_ = 1.372).

#### Gamma-Band

**FIGURE 5:**
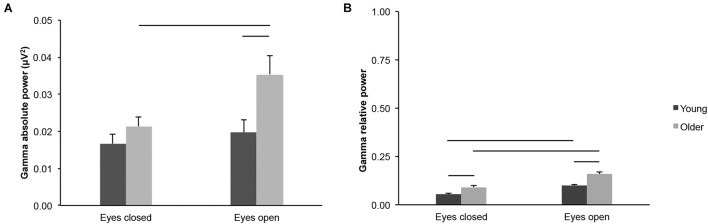
Gamma-band activity measured across the scalp in healthy 20–30-year-old (Young; dark gray bars) and 65–75-year-old (Older; light gray bars) adults. **(A)** Gamma absolute power. **(B)** Gamma relative power. Average ± SEM. Horizontal bars indicate statistically significant differences between age groups or eye conditions (*p* < 0.05).

We found age-group differences for gamma-band activity measured across the scalp. Gamma absolute power was higher in older adults than in young adults in the eyes open condition, but not in the eyes closed condition (Figure 5A; age groups: *F*_(1,43)_ = 4.552, *p* = 0.039, young < older, η*^2^_*p*_* = 0.096; age groups × eye conditions: *F*_(1,43)_ = 8.003, *p* = 0.007, η*^2^_*p*_* = 0.157; eyes open, young < older, *t*_(43)_ = 2.474, *p* = 0.017, *d*_*s*_ = 0.739; eyes closed, *t*_(43)_ = 1.301, *p* = 0.200, *d*_*s*_ = 0.389). Gamma relative power was higher in older adults than in young adults in both eye conditions (Figure 5B; age groups: *F*_(1,43)_ = 14.336, *p* < 0.001, young < older, η*^2^_*p*_* = 0.250; eyes open, *t*_(43)_ = 3.789, *p* < 0.001, *d*_*s*_ = 1.132; eyes closed, *t*_(43)_ = 3.233, *p* = 0.002, *d*_*s*_ = 0.966), despite a significant interaction between age groups x eye conditions (*F*_(1,43)_ = 4.909, *p* = 0.032, η*^2^_*p*_* = 0.102).

Gamma absolute power was higher in the eyes open condition than in the eyes closed condition for older adults, but not for young adults (Figure 5A; eye conditions: *F*_(1,43)_ = 19.124, *p* < 0.001, η*^2^_*p*_* = 0.308; interaction see above; young: open > closed: *t*_(20)_ = 1.752, *p* = 0.095, *d*_*z*_ = 0.382; older: open > closed *t*_(23)_ = 4.230, *p* < 0.001, *d*_*z*_ = 0.863). Gamma relative power was higher in the eyes open condition than in the eyes closed condition for both age groups (Figure 5B; eye conditions: *F*_(1,43)_ = 113.266, *p* < 0.001, open > closed, η*^2^_*p*_* = 0.725; young: open > closed, *t*_(20)_ = 7.899, *p* < 0.001, *d*_*z*_ = 1.724; older: open > closed, *t*_(23)_ = 7.938, *p* < 0.001, *d*_*z*_ = 1.620).

#### Total Power

**FIGURE 6:**
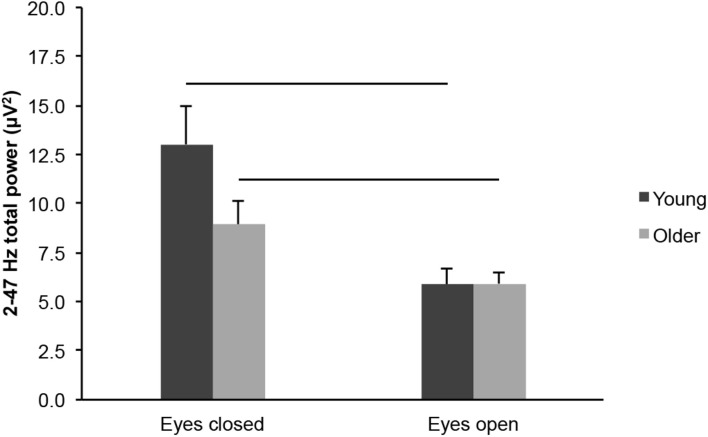
Total EEG power (2–47 Hz) measured across the scalp in healthy 20–30-year-old (Young; dark gray bars) and 65–75-year-old (Older; light gray bars) adults. Average ± SEM. Horizontal bars indicate statistically significant differences between eye conditions (*p* < 0.05).

We did not find differences in total EEG power (2–47 Hz) between young and older adults across eye conditions (Figure 6; age groups: *F*_(1,43)_ = 1.838, *p* = 0.182, η*^2^_*p*_* = 0.041), but there was an interaction between age groups and eye conditions (*F*_(1,43)_ = 5.528, *p* = 0.023, η*^2^_*p*_* = 0.114). There was no difference between age groups in the eyes open condition (*t*_(43)_ = 0.063, *p* = 0.950, *d*_*s*_ = 0.019), whereas the difference between age groups was nearly significant in the eyes closed condition (*t*_(43)_ = 1.823, *p* = 0.075, *d*_*s*_ = 0.545). Total EEG power was lower in the eyes open condition than in the eyes closed condition, for both age groups (Figure 6; eye conditions: *F*_(1,43)_ = 33.200, *p* < 0.001, open < closed, η*^2^_*p*_* = 0.436; young: open < closed, *t*_(20)_ = 4.288, *p* < 0.001, *d*_*z*_ = 0.936; older: open < closed, *t*_(23)_ = 3.896, *p* = 0.001, *d*_*z*_ = 0.795).

### Allocentric Spatial Working Memory Performance

**FIGURE 7:**
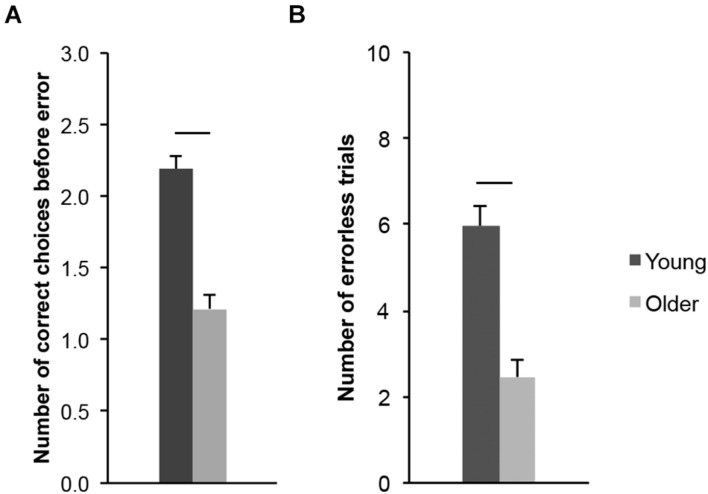
Allocentric spatial working memory performance of healthy 20–30-year-old (Young; dark gray bars) and 65–75-year-old (Older; light gray bars) adults. **(A)** Number of correct choices before erring (CBE). **(B)** Number of errorless trials (NET). Average ± SEM. Horizontal bars indicate statistically significant differences between age groups (*p* < 0.05).

We first analyzed the number of goal locations that young (20–30-year-old) and older (65–75-year-old) adults visited before making an error (CBE; Figure 7A; a proxy for memory capacity). Older adults made fewer correct choices before erring (*M* = 1.22, SD = 0.50) than young adults (*M* = 2.19, SD = 0.44; *t*_(43)_ = 6.951, *p* < 0.001, *d*_*s*_ = 2.077). We then considered the number of errorless trials (NET; Figure 7B; a measure of perfect memory performance). Older adults performed fewer errorless trials (*M* = 2.46, SD = 1.86) than young adults (*M* = 5.95, SD = 2.18; *t*_(43)_ = 5.798, *p* < 0.001, *d*_*s*_ = 1.732). Altogether, these measures revealed a lower spatial working memory performance in older adults, consistent with data in the literature regarding age-related differences in working memory performance (see above) and previous findings from our laboratory (Klencklen et al., 2017a,b).

### Principal Component and Multiple Regression Analyses

To determine whether some resting-state EEG signatures may correlate with allocentric spatial working memory performance in young and older adults, we performed a Principal Component Analysis (PCA) including the 30 EEG variables reported above (Supplementary Material 5). Horn’s Parallel Analysis revealed four components, which explained 80.52% of the total variance. The EEG variables that contributed to each of the four components retained in the PCA analysis are listed in Table 2. A table reporting the correlations between EEG variables and selected PCA components is provided in Supplementary Material 6.

**TABLE 2:** EEG measures that contributed to the four components retained from the PCA analysis.

	**Eyes closed condition**			**Eyes open condition**			
**Component 1**							
Theta	AP Power	Abs Power		AP Power	Abs Power		
Alpha	Peak power	Abs Power	Rel Power	Peak power	Abs Power		
Beta	AP Power	Abs Power		AP Power	Abs Power		
Gamma	Rel Power						
2–47 Hz	Power			Power			
**Component 2**							
Theta				Rel Power			
Alpha				Rel Power			
Beta	Rel Power			AP Frequency	AP Power	Abs Power	Rel Power
Gamma	Abs Power	Rel Power		Abs Power	Rel Power		
1–47 Hz							
**Component 3**							
Theta	AP Frequency			AP Frequency			
Alpha	AP Frequency			AP Frequency	Rel Power		
Beta							
Gamma							
1–47 Hz							
**Component 4**							
Theta				AP Frequency			
Alpha							
Beta				AP Frequency			
Gamma							
1–47 Hz							

*AP, averaged peak; Abs, absolute; Rel, relative.*

**FIGURE 8:**
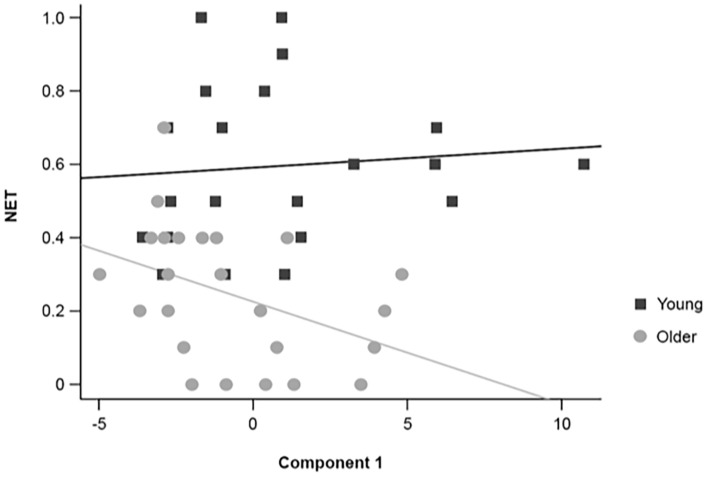
Graphical representation of the individual scores and regression lines between the first EEG component extracted with the PCA and NET for healthy 20–30-year-old (Young; dark gray squares) and 65–75-year-old (Older; light gray circles) adults.

Multiple regression analyses revealed that some PCA components accounted partially for working memory performance (Table 3), as measured by the number of errorless trials (NET). Moreover, the partial regression coefficient between the first EEG component and NET differed between young and older adults (4-component model: *t*_(35)_ = −2.400, *p* = 0.022; simplified model: *t*_(37)_ = −2.313, *p* = 0.026; Table 3). In addition, the slope of the regression line between the first EEG component and NET (Figure 8) was different from zero in older adults (slope = −0.424, SE = 0.181; *t*_(37)_ = −2.344, *p* = 0.025), but not in young adults (slope = 0.129, SE = 0.142; *t*_(37)_ = 0.911, *p* = 0.368).

**TABLE 3:** Multiple regression analyses investigating the possible links between the EEG components extracted using PCA and allocentric spatial working memory performance (CBE, NET).

	**CBE**			**NET**		
***4-component model***	**β**	***t***	***p***	**β**	***t***	***p***
Age group	−1.14	−3.935	**<0.001**	−0.98	−3.301	**0.002**
Comp. 1	−0.03	−0.221	0.827	−0.10	−0.780	0.441
Comp. 2	−0.27	−1.927	0.062	−0.30	−2.114	**0.042**
Comp. 3	−0.05	−0.445	0.659	0.01	0.077	0.939
Comp. 4	0.19	1.601	0.118	0.27	2.156	**0.038**
Age × Comp.1	−0.43	−1.802	0.080	−0.59	−2.400	**0.022**
Age × Comp.2	0.33	1.170	0.250	0.14	0.476	0.637
Age × Comp.3	0.06	0.225	0.824	0.30	1.196	0.240
Age × Comp.4	−0.26	−1.087	0.285	−0.24	−0.968	0.340
*F*_(9,35)_		7.26	**<0.001**		6.81	**<0.001**
Adj. R^2^		0.56			0.54	
*R* ^2^		0.65			0.64	

***Simplified model***	**β**	***t***	***p***	***β***	***t***	***p***

Age group	−1.18	−4.636	**<0.001**	−1.06	−3.747	**<0.001**
Comp. 1	−0.01	−0.085	0.933	−0.15	−1.335	0.190
Comp. 2	−0.22	−1.753	0.088	−0.28	−2.046	**0.048**
Comp. 3	–	–	–	−0.03	−0.222	0.825
Comp. 4	0.22	2.013	0.051	0.23	1.976	0.056
Age × Comp. 1	−0.42	−1.857	0.071	−0.55	−2.313	**0.026**
Age × Comp. 3	–	–	–	0.35	1.421	0.164
Age × Comp. 4	−0.32	−1.486	0.145	–	–	–
*F*_(6,38)_, *F*_(7,37)_		10.96	**<0.001**		8.77	**<0.001**
Adj. R^2^		0.58			0.55	
*R* ^2^		0.63			0.62	

The difference between age groups in the partial regression coefficient between the first EEG component and CBE just failed to reach the predefined level of statistical significance (4-component model: *t*_(35)_ = −1.802, *p* = 0.080; simplified model: *t*_(38)_ = −1.857, *p* = 0.071; Table 3). The slope of the regression line between the first EEG component and CBE was not different from zero in young adults (slope = 0.201, SE = 0.139; *t*_(38)_ = 1.441, *p* = 0.158) or older adults (slope = −0.220, SE = 0.181; *t*_(38)_ = −1.220, *p* = 0.230). It is interesting to note that the interaction between the first EEG component and age groups is retained in all simplified models, even if it is not considered to be statistically significant in subsequent analyses.

## Discussion

Regarding the first aim of the study, our data confirmed that age-related differences in resting-state brain activity depend on the conditions of EEG recordings (eyes open versus eyes closed) and the parameters used to define brain activity in different frequency bands, and provide a coherent framework in which to consider the results of previous studies. Accordingly, we found a number of consistent differences in resting-state brain activity between 20–30-year-old and 65–75-year-old healthy adults: older adults exhibited lower theta-band frequency and absolute power, lower alpha-band frequency and absolute power, higher beta-band relative power, and higher gamma-band relative power.

Regarding the second aim of the study, using PCA and multiple regression analyses, we found that the relationships between some resting-state EEG parameters and allocentric spatial working memory performance differed between young and older adults. The first extracted EEG component, which represented mainly theta, alpha and beta powers, was correlated to errorless working memory performance in older adults, but not in young adults. These results suggest potential age-related differences in the neurobiological bases of allocentric spatial working memory performance. However, EEG recording of resting-state brain activity could not be used as a reliable biomarker to predict *individual* spatial working memory performance in either young or older participants.

### Age-Group Differences in Resting-State Brain Activity

#### Theta-Band

We found lower theta-band frequency and power in older adults, as compared to young adults, in both eye conditions. Our findings are consistent with previous research showing an overall age-related reduction of theta activity (Supplementary Material 1; Matousek et al., 1967; Duffy et al., 1984, 1993; Breslau et al., 1989; Williamson et al., 1990; Hartikainen et al., 1992; Widagdo et al., 1998; Cummins and Finnigan, 2007; Volf and Gluhih, 2011; Vysata et al., 2012; Van de Vijver et al., 2014; Reichert et al., 2016; Barry and De Blasio, 2017; Trammell et al., 2017). Some studies also reported that theta relative power was relatively stable across ages in the eyes closed condition (Penttila et al., 1985; Giaquinto and Nolfe, 1986; Hartikainen et al., 1992; Widagdo et al., 1998; Reichert et al., 2016; Ponomareva et al., 2017). This is consistent with our findings that the age-group difference in theta relative power is less prominent in the eyes closed condition.

In contrast, some studies have failed to find lower theta activity in older adults at rest. In particular, Hartikainen et al. (1992) showed a reduction of theta (4.1–7.3 Hz) absolute amplitude, but they did not find changes of theta absolute power with age. We have identified four potential explanations which may account for the contradictory findings of Hartikainen et al. (1992). First, power measures (μV^2^) introduce a non-linear variation to amplitude measures (μV), which may be less sensitive to small changes in signal or signal to noise ratios. Second, of their 52 elderly participants, 31 complained of mild cognitive impairments that the authors eventually deemed trivial. As resting-state theta activity has been reported to increase in MCI or Alzheimer’s disease (Vecchio et al., 2013), it may not be surprising to observe no age-related differences in theta activity in a population of older adults comprising healthy and mildly cognitively impacted individuals. In our study, we recruited healthy, independently living elderly with no subjective complaints. Third, Hartikainen et al. (1992) selected participants by a visual evaluation of their EEG and considered as normal an alpha relative power of at least 50%. Inspection of our data revealed that many of our participants did not reach this criterion; only 12/24 older adults and 14/21 young adults exhibited an alpha relative power of at least 50% in the eyes closed condition. As our participants were healthy adults, it is reasonable to think that such differences in alpha relative power are part of the normal variation in the population. Fourth, their study was limited to two temporo-occipital recording sites.

Kononen and Partanen (1993) did not find a correlation between theta (4.15–7.32 Hz) absolute amplitude and age in a population between 23 and 80 years of age in the eyes closed condition, but they found a positive correlation between theta absolute amplitude and age in the eyes open condition. Similar to the study of Hartikainen et al. (1992), this study was limited to four recording sites (temporo-occipital and temporo-central sites). Moreover, they selected only participants whose EEG was considered normal, but no specific information was provided to define normality.

Gaal et al. (2010) did not find changes in theta (4–8 Hz) power with age. They recruited 18–35-year-olds and 60–75-year-olds from the general population and controlled for IQ differences. Their population had no history of neurological or psychiatric disorders. However, their power values were much higher than in the present study and seemed to decrease with age. Moreover, although global power was lower in the older group, a frequency × age interaction was only reported for delta power. Finally, Hartikainen et al. (1992), Kononen and Partanen (1993), and Gaal et al. (2010) also reported contradictory results for alpha and beta activity, which will not be discussed further (Supplementary Materials 1–3).

Fan et al. (2014) reported no difference between theta power (4–8 Hz) at rest with the eyes closed between older (mean age: 74.24; *N* = 9) and young (mean age: 35.14; *N* = 16) adults, but no data were shown. Their young adult group was 10 years older than in the current study, so it is interesting to note that most studies comparing older adults with young adults around 30 years of age failed to show age-related differences in theta activity during eyes closed recordings, suggesting that theta activity might already be decreasing by 30 years of age (Penttila et al., 1985; Hartikainen et al., 1992; Widagdo et al., 1998; Ponomareva et al., 2017).

Oken and Kaye (1992) reported a positive correlation between age and theta relative power (4.25–8 Hz) in a population of healthy adults between 20 and 99 years of age. Although their population was screened for dementia and cognitive impairments, their correlation might nonetheless be due to the inclusion of extremely old participants (80 to 99 years of age), as suggested by the correlation between ERP components’ amplitude and age in their study. Moreover, although recordings from 14 sites were collected in their study, only two recording sites (occipito-parietal) were used for their analyses because half of the older participants exhibited excessive movement artifact over temporal regions.

Finally, Pollock et al. (1990) reported no correlation between age and theta absolute amplitude in a population of healthy adults between 56 and 76 years of age. The absence of correlation might be due to the smaller age range and the strict statistical analysis. Williamson et al. (1990) reported weak negative correlations between age and theta absolute power, at 4 of 8 recording sites, in a population of healthy men between 65 and 81 years of age.

In sum, the majority of data, including our own, is consistent in showing that in mentally healthy and independently living individuals, resting-state brain activity in the theta frequency band decreases between age 20 and age 70 in both the eyes open and the eyes closed conditions.

##### Theta-Band Function

The functional significance of theta modulation with age is still unclear. Theta-band activity has been shown to be involved in brain plasticity (for a review see Kahana et al., 2001), memory encoding, retrieval, LTP and working memory processes (Klimesch, 1999; Ward, 2003; Werkle-Bergner et al., 2006; Sauseng et al., 2010; Ekstrom and Watrous, 2014). However, studies attempting to clarify the link between theta-band activity at rest and memory are sparse and contrasting. Nevertheless, data suggest that the link between theta-band activity, either while performing a task or while at rest, and memory processes seems to evolve with age. For example, during encoding of a 3D virtual maze, theta power has been shown to be positively correlated with subsequent retrieval of the learned path in young individuals (21–29 years of age), whereas in older adults (60–73 years of age) theta relative power was reduced, spatial memory performance impaired, and the two measures were no longer correlated, suggesting a difference in the neural dynamics subserving memory encoding (Lithfous et al., 2015). Similarly, at rest, Vlahou et al. (2014) showed that MEG theta-band (4–7.5 Hz) activity was differentially associated with cognitive performance in young (21–29 years of age) and older adults (60–73 years of age). Participants had to perform two Trail Making tests, during which they had to connect numbers (1, 2, 3, …; test A), or alternating numbers and letters (1, A, 2, B, …; test B) in an ascending manner as quickly as possible. Vlahou et al. (2014) showed that performance on both tests was negatively correlated with age. In addition, theta power at rest was positively correlated with subsequent test B performance in older adults, but not in young adults, suggesting a difference in the neural dynamics subserving executive functions between young and older individuals.

#### Alpha-Band

We found lower resting-state alpha-band activity parameters in older adults: specifically alpha peak frequency and alpha absolute power, and a marginal difference of alpha peak power and relative power, across eye conditions. Alpha peak frequency values in the current study fall within the ranges previously reported in the eyes closed condition (Supplementary Material 2). We found an average individual alpha peak frequency of 10.38 Hz in young adults and 9.56 Hz in older adults. Klimesch (1999) estimated alpha peak frequency at 10.89 Hz around 20 years of age, and at 8.24 Hz around 70 years of age. Lodder and van Putten (2011) found that the frequency of the posterior dominant rhythm was 10.21 Hz between 20 and 40 years of age, and 9.37 Hz between 60 and 80 years of age. Scally et al. (2018) found an individual alpha peak frequency of 10.04 Hz in young adults and 8.78 Hz in older adults. Most other studies also reported a decrease in alpha peak frequency with age (Oken and Kaye, 1992; Chiang et al., 2011; Lodder and van Putten, 2011; Trammell et al., 2017; Knyazeva et al., 2018; Scally et al., 2018). Reichert et al. (2016) did not report significant age-group differences in alpha peak frequency at electrode Pz. However, their graphic representation revealed a peak frequency of about 10 Hz for young adults and about 9 Hz (mean peak frequency of double peaks) in older adults, which correspond to the values reported previously and in the current study. Similarly, although Duffy et al. (1984) found no correlation between age and individual peak frequency in a population of 30- to 80-year-old healthy adults, they reported a mean peak frequency of 9.79 Hz in 30–40-year-old adults and a mean peak frequency of 9.03 Hz in 70–80-year-old adults [see also (Penttila et al., 1985; Hartikainen et al., 1992)].

Similar to our findings, Scally et al. (2018) found a lower individual alpha peak frequency in older adults [see also (Gaal et al., 2010); but see discussion above], and no differences of individual peak power. These authors suggested that although alpha peak frequency oscillations are slowing with age, perhaps due to age-related changes in the thalamus (Cherubini et al., 2009), the output of the dominant alpha generators might be intact. However, we found that alpha absolute power was lower in older adults across eye conditions, suggesting a difference in the functional dynamics of alpha generators. Indeed, a number of other studies have shown that alpha absolute power or amplitude is reduced with age in both the eyes closed condition (Breslau et al., 1989; Volf and Gluhih, 2011; Vysata et al., 2012; Fan et al., 2014; Barry and De Blasio, 2017; Scally et al., 2018) and the eyes open condition (Barry and De Blasio, 2017), but see Hartikainen et al. (1992), Kononen and Partanen (1993) and Gaal et al. (2010) discussed above. Volf and Gluhih (2011) found no age-related differences in alpha absolute power in the eyes open condition. In our study, we found a greater difference between age groups in alpha absolute power in the eyes closed condition, and a marginal interaction between eye conditions and age groups suggesting that distinct processes underlying alpha-band activity in eyes open versus eyes closed conditions may be differentially affected by age. Reduced alpha-band activity when the eyes are open as compared to when the eyes are closed has been associated with an increase in arousal when the eyes are open (Klimesch, 1999 for a review), which might reflect thalamo-cortical uncoupling supporting visual processing as proposed by Barry and De Blasio (2017).

Finally, we did not find age-group differences in alpha relative power in either eye condition, although a trend toward a decrease was present. Most studies show no age-related differences in alpha relative power, with either the eyes closed (Penttila et al., 1985; Ponomareva et al., 2017; Trammell et al., 2017) or the eyes open (Widagdo et al., 1998; Reichert et al., 2016); or a slightly lower alpha relative power in the eyes closed condition (Widagdo et al., 1998; Vysata et al., 2012). Reichert et al. (2016) found a lower alpha relative power in older adults in the eyes closed condition, but these authors used a fixed alpha-band of 10–12 Hz, which corresponds to the mean alpha peak in young individuals and is higher than the mean alpha peak observed in older individuals. This choice likely inflated the difference between young and older adults in their study.

In sum, our data, as well as previous data, indicate that in mentally healthy and independently living individuals alpha absolute activity is lower in 65–75-year-old adults than in 20–30-year-old adults, particularly in the eyes closed condition. Alpha relative activity seems to be relatively stable across ages, with perhaps a slight tendency to be lower in older adults.

##### Alpha-Band Function

Alpha-band activity has been linked to attention and inhibitory processes (Klimesch et al., 2007, 2011; Jensen and Mazaheri, 2010). It is well known that both gray matter and white matter undergo significant atrophy with age, particularly in frontal regions, affecting functional connectivity of the brain (Lockhart and DeCarli, 2014 for a review). White matter atrophy has been associated with altered executive functions (i.e., processing speed, working memory, inhibition, task switching) and episodic memory performance (Kennedy and Raz, 2009). Interestingly, some studies have shown that an increase in alpha activity is associated with a decrease in BOLD cortical signal (e.g., Goldman et al., 2002) and coherence (e.g., Tagliazucchi et al., 2012), prompting suggestions that alpha activity might therefore reflect cortical inactivation (Goldman et al., 2002). The lower alpha activity in older adults found in the current and previous studies might therefore reflect alterations in cortical inhibitory processes, which might impact working memory performance. For example, Klimesch et al. (2003) found that alpha suppression (the reduction of alpha activity between pre-stimuli baseline and stimuli presentation) is an important factor contributing to success in mental rotation tasks, and that the greater the alpha activity is at baseline, the greater the suppression will be during stimuli presentation (Doppelmayr et al., 1998). A lower alpha baseline activity in older adults might therefore reduce the level of alpha suppression between baseline and task performance and thus reflect a general reduction in executive functions and memory processing abilities in older adults (see also next section).

#### Beta-Band

We found a higher beta relative power in older adults in both eye conditions (no age-group differences for other beta parameters). In the few studies that have reported age-related differences in beta activity at rest (Supplementary Material 3), beta relative power has been mostly reported to be higher in older adults, both with the eyes closed (Widagdo et al., 1998; Vysata et al., 2012; Ponomareva et al., 2017) and the eyes open (Duffy et al., 1984, 1993; Widagdo et al., 1998). One study did not find a correlation between age and beta relative power in a population of 20- to 99-year-olds (Oken and Kaye, 1992). Note again, however, the presence of extremely old adults in this sample. Moreover, although recordings were made from 14 sites in their study, only two occipito-parietal recording sites were used for analysis because half of the older participants had excessive movement artifact over temporal regions.

We did not find reliable age-group differences in beta absolute power in either eye condition. Some studies previously reported that beta absolute activity is higher in older adults, with either the eyes open (Duffy et al., 1984; Volf and Gluhih, 2011; Barry and De Blasio, 2017), or the eyes closed (Fan et al., 2014; Barry and De Blasio, 2017). Other studies reported that with the eyes closed beta absolute activity was either lower in older adults (Breslau et al., 1989; Vysata et al., 2012), or did not differ between age groups (Volf and Gluhih, 2011). Although it is difficult to reach a consensus due to the few studies reporting parameters of beta activity across ages, our data, as well as previous data, suggest that in mentally healthy and independently living individuals beta relative power is higher in 60–70-year-old than in 20–30-year-old individuals, in both eye conditions.

##### Beta-Band Function

Beta activity has been linked to motor control and inhibitory activity within motor cortices (Rossiter et al., 2014; Heinrichs-Graham and Wilson, 2016). Beta activity is enhanced in Parkinson’s disease and has been proposed to reflect an abnormal persistence of the status quo (no expectancy for changes to occur) and a deterioration of flexible behavioral and cognitive control (Engel and Fries, 2010). Barry and De Blasio (2017) hypothesized that higher beta activity might reflect the addition of processing resources needed to maintain reactivity to environmental changes in older adults.

#### Gamma-Band

We found a higher gamma-band absolute power in older adults, but only in the eyes open condition. Our data are consistent with those of Volf and Gluhih (2011), showing a higher gamma absolute power in older adults in the eyes open but not in the eyes closed condition. Fan et al. (2014), using a wider gamma-band (30–100 Hz) as compared to our study (30–47 Hz) or the Volf and Gluhih (2011) study (30–50 Hz), also found higher gamma absolute power in 74-year-old adults as compared to 35-year-olds. In contrast, Vysata et al. (2012), who recorded eyes closed resting-state EEG only, found a mild decrease of gamma absolute power (30–60 Hz) with age. Their study analyzed the EEG recordings of 17,722 truck drivers between 20 and 70 years of age, and a regression analysis identified a 0.18% yearly decline of gamma absolute power. However, these authors also found an increase of gamma relative power with age, which is consistent with our current findings across eye conditions. Our data, as well as the very few previous studies on age-related differences in gamma therefore suggest that in mentally healthy and independently living individuals gamma relative power (30–50 Hz) is higher in 70-year-old than in 20-year-old individuals.

##### Gamma-Band Function

Several cognitive functions have been linked to gamma-band activity, like working memory and attentional processes (Jensen et al., 2007; Benchenane et al., 2011; Miller et al., 2018). Interestingly, it has been proposed that synchronization in the gamma range is fundamental for selecting and integrating information in distributed networks (Senkowski et al., 2008; Fries, 2009). Similar to beta-band activity, higher gamma-band activity in older adults might reflect the need for more resources for multisensory integration (Senkowski et al., 2009).

### Resting-State Brain Activity and Working Memory

When taken together, the findings from our current study and the previously published work of others provides a relatively coherent view of differences in resting-state brain activity between young and older healthy adults. Lower theta- and alpha-band frequencies and absolute powers, and higher beta- and gamma-band relative powers are consistently observed in older adults. However, the link between specific resting-state EEG parameters and specific cognitive capacities, and working memory performance in particular, is more complex (Table 1). Whereas some studies have identified correlations between resting-state brain activity in certain frequency bands and memory performance, other studies have failed to do so. Moreover, although limited correlations may exist between resting-state brain activity and some cognitive functions, the value of such correlations to make predictions about individual subjects that would be useful for diagnostic purposes in clinical settings is difficult to defend based on currently available data.

Nonetheless, combined EEG parameters (extracted using PCA) were differentially correlated to spatial working memory performance in young and older adults. Indeed, we found a significant partial regression coefficient between the first EEG component and NET that differed between young and older adults. These analyses suggest that different functional networks may contribute to working memory processes in older versus young individuals. Accordingly, it has been shown that resting-state brain functional dynamics change with age. Vysata et al. (2014) showed a lower coherence in several frequency bands in older adults suggesting a reduction of cortical connectivity with age. Several resting-state fMRI studies have confirmed this hypothesis, showing a decrease in connectivity with age in the resting brain (for a review see Ferreira and Busatto, 2013). Petti et al. (2016) studied 71 participants between 20 and 63 years of age, with EEG and advanced signal processing methods (Partial Directed Coherence). They showed that network communication and global strength tended to decrease with age. These authors suggested that brain functional organization tends to become less organized and more random with aging (Petti et al., 2016). Knyazev et al. (2015) using graph-theoretical analysis on the EEG data of 76 young (18–35 years) and 70 older (51–80 years) participants showed a lower connectivity in beta- and gamma-band networks in older adults, also leading to the suggestion that brain networks become more random with age (Knyazev et al., 2015).

Interestingly, and particularly germane to the current study, in rats resting-state functional connectivity exhibits qualitative changes during aging even in the absence of cognitive decline (Ash et al., 2016). Whereas older rats with spatial memory impairments in a Morris Water Maze exhibit a distinct network signature as compared to young rats, older rats with preserved spatial memory abilities in the Morris Water Maze exhibit a reduced functional connectivity in the same network as observed in young rats. These data suggest that successful aging is associated with adaptive remodeling, and not simply the maintenance of youthful network dynamics (Ash et al., 2016). See also Tomas Pereira et al. (2015) for similar conclusions; but see Coquelet et al. (2017) for recent data in favor of the brain maintenance theory for successful aging.

In humans, Rondina et al. (2016) found similar results. They studied 16 young (mean age: 24.8 years) and 16 older (mean age: 65.9 years) adults using MEG recordings during a visuo-spatial relational binding task. Participants had to recollect the relative position of two objects that had been previously presented separately at different positions on a computer screen. Although older adults exhibited task performance and hippocampal volume similar to young adults, pre-stimulus theta relative power was lower and beta relative power higher in the older participants, as compared to the young. Interestingly, the authors also found both a positive correlation between pre-stimulus relative theta power and hippocampal volume (*r* = 0.52, *p* < 0.05), and a negative correlation between pre-stimulus beta relative power and hippocampal volume (*r* = −0.70, *p* < 0.005) in young adults, but not in older adults. Their study thus showed differences in resting-state brain dynamics in older individuals in absence of memory decline or hippocampal atrophy. These findings support the idea that different functional networks may subserve working memory functions in older versus young individuals.

In sum, the current study provides the first description of resting-state EEG brain activity associated with the performance of a hippocampus-dependent spatial working memory task in humans that is homologous to the memory tasks used in rodents. We found that the relationship between resting-state EEG activity and allocentric spatial working memory performance differed between young and older adults, suggesting the contribution of different functional networks at different ages. Our findings are largely consistent with data in rodents and humans showing qualitative changes of resting-state brain connectivity during normal aging.

## Conclusion

Our study has found a number of age-group differences in resting-state brain activity in different recording conditions, which are consistent with previous results. We confirmed that age-group differences in resting-state EEG activity depend largely on the recording conditions and the specific parameters considered in the analyses. Nevertheless, lower theta-band and alpha-band frequencies and absolute powers, and higher beta-band and gamma-band relative powers were overall observed in healthy older adults, as compared to healthy young adults. In addition, using a principal component analysis, we found that the first extracted EEG component, which represented mainly theta, alpha and beta powers, was linked to spatial working memory performance in older adults, but not in young adults. However, resting-state EEG activity could not be used as a reliable biomarker to predict the spatial working memory performance of individual subjects. Together with previous studies in rats and humans, our current findings suggest that the neurobiological bases of working memory performance may differ between healthy young and older adults.

## Data Availability Statement

The datasets presented in this study can be found in online repositories. The names of the repository/repositories and accession number(s) can be found below: All EEG recording data are freely available at: 10.5281/zenodo.3875159.

## Ethics Statement

The studies involving human participants were reviewed and approved by the Cantonal Ethics Committee (Vaud, Switzerland, Protocol No 384/15). The participants provided their written informed consent to participate in this study.

## Author Contributions

PL, PBL, AJ, and GK contributed to the conception and design of the study. AJ, GK, and PR collected the data. AJ, PR, J-PA, and PL analyzed the data and performed the statistical analyses. AJ and PL wrote the first draft of the manuscript. All authors contributed to manuscript revision, read, and approved the submitted version.

## Conflict of Interest

The authors declare that the research was conducted in the absence of any commercial or financial relationships that could be construed as a potential conflict of interest.

## Publisher’s Note

All claims expressed in this article are solely those of the authors and do not necessarily represent those of their affiliated organizations, or those of the publisher, the editors and the reviewers. Any product that may be evaluated in this article, or claim that may be made by its manufacturer, is not guaranteed or endorsed by the publisher.

## Supplementary Material

Supplementary Material 1 Studies on age-group changes in theta activity: age ranges considered, EEG parameters extracted and main findings.

Supplementary Material 2 Studies on age-group changes in alpha activity: age ranges considered, EEG parameters extracted and main findings.

Supplementary Material 3 Studies on age-group changes in beta activity: age ranges considered, EEG parameters extracted and main findings.

Supplementary Material 4 Studies on age-group changes in gamma activity: age ranges considered, EEG parameters extracted and main findings.

Supplementary Material 5 Individual data used to perform the statistical analyses reported in the main manuscript.

Supplementary Material 6 Correlations between EEG parameters and the four selected components of the PCA analysis.
