# Supplementary material for: Age-Related Differences in Resting-State EEG and Allocentric Spatial Working Memory Performance
Source: Front Aging Neurosci. 2021 Nov 4;13:704362. doi: 10.3389/fnagi.2021.704362 (PMC8600362; doi:10.3389/fnagi.2021.704362)
Supplement: Supplementary Material 1 — Studies on age-group changes in theta activity: age ranges considered, EEG parameters extracted and main findings. [file Table_1.docx]

| **Supplementary Material 1.** Studies on age-related changes in theta-band activity: age ranges considered, EEG parameters extracted and main findings. | | | | | |
| --- | --- | --- | --- | --- | --- |
| **Study** | **Mean age and/or age range** | **Frequency band** | **Parameters extracted** | **Results eyes open** | **Results eyes closed** |
| *Duffy et al., 1984* | 30-80 (males only) | 4-7.75Hz | absolute amplitude (μV)  relative amplitude | 🡾, correlation with age  🡾, correlation with age | -  - |
|  |  |  |  |  |  |
| *Cummins & Finnigan, 2007* | 22 vs 68 | 4.88-6.84Hz | absolute power (μV^2^) | 🡾 | - |
|  |  |  |  |  |  |
| *Volf & Gluhih, 2011* | 22 vs 65 | 4-8Hz | absolute power (μV^2^/Hz; log transformed) | 🡾 | 🡾 |
|  |  |  |  |  |  |
| *Barry & De Blasio, 2017* | 20 vs 68 | 4-7.5Hz | absolute amplitude (μV) | 🡾 | 🡾 |
|  |  |  |  |  |  |
| *Van de Vijver et al., 2014* | 22 vs 69 | 3-7Hz older / 4-8Hz young | absolute power (dB) | 🡾, at FCz  **ns** at other sites | - |
|  |  |  |  |  |  |
| *Reichert et al., 2016* | 25 vs 48 vs 68 | 4-8Hz | relative power (μV^2^; log transformed) | 🡾, 25>48≅68 | **ns** |
|  |  |  |  |  |  |
| *Widagdo et al., 1998* | 29 vs 73 | 4-7.5Hz | relative power (-) | 🡾 | **ns** |
|  |  |  |  |  |  |
| *Duffy et al., 1993* | 30-80 | 4-7.5Hz | relative amplitude (log transformed) | 🡾, ANOVA (groups by decades) | - |
|  |  |  |  |  |  |
| *Vysata et al., 2012* | 20-70 | 4.7.5Hz | absolute power (μV^2^/Hz)  relative power (μV^2^/Hz) | - | 🡾, linear regression  🡾, linear regression |
|  |  |  |  |  |  |
| *Breslau et al., 1989* | 23 vs 70 | 4.7-7.8Hz | absolute amplitude (μV) | - | 🡾 |
|  |  |  |  |  |  |
| *Hartikainen et al., 1992* | 31 vs 53 vs 71 | 4.1-7.3Hz | absolute amplitude (μV)  absolute power (μV^2^)  relative amplitude and power | -  - | 🡾, 31 ≅ 53 > 71 🡾, correlation with age  **ns**  **ns** |
|  |  |  |  |  |  |
| *Trammell et al., 2017* | 21 vs 73 | 4-8Hz | relative power (-) | - | 🡾, correlations with age at Fz and Cz  **ns**, correlations with age at other sites |
|  |  |  |  |  |  |
| *Pentilla et al., 1985* | 33 vs 73 | 4.15-7.32Hz | relative power (-) | - | **ns** |
|  |  |  |  |  |  |
| *Ponomareva et al., 2017* | 36 vs 62 | 4-7.99Hz | relative power (log transformed) | - | **ns** |
|  |  |  |  |  |  |
| *Gaal et al., 2010* | 22 vs 67 | 4-8Hz | absolute power (μV^2^/Hz) | **ns** | **ns** |
|  |  |  |  |  |  |
| *Kononen & Partanen, 1993* | 23-80 | 4.11-7.32Hz | absolute amplitude (μV, log transformed) | 🡽, correlation 23-80yrs (posterior)  **ns**, correlation 20-60yrs (posterior)  🡽, correlation 60-80yrs (posterior) | **ns** |
|  |  |  |  |  |  |
| *Fan et al., 2014* | 35 vs 74 | 4-8Hz | absolute power (-) | - | 🡾 |
|  |  |  |  |  |  |
| *Oken & Kaye, 1992* | 20-99 (<65 vs >65) | 4.25-8Hz | relative power (log transformed) | - | 🡽, posterior sites |
|  |  |  |  |  |  |
| *Matousek et al., 1966* | 17-64 | 3.5-7.5Hz | absolute amplitude (μV/sec, log transformed) | - | 🡾, correlation with age |
|  |  |  |  |  |  |
| *Giaquinto and Nolfe, 1986* | 49 vs 71 | 4-7.75Hz | relative power (-) | - | **ns** |
|  |  |  |  |  |  |
| *Pollock et al., 1990* | 56-76 | 4.3-7.8Hz | absolute amplitude (μV , log transformed) | **ns,** correlation with age | **ns,** correlation with age |
|  |  |  |  |  |  |
| *Williamson et al., 1990* | 65-81 | 4-8Hz | absolute power (-) | - | 🡾, correlation (males only) |
|  |  |  |  |  |  |
| **Abbreviations and symbols**  Parameters extracted: units reported in each study provided in parenthesis; (-) units not described in the study.  Results: 🡾 decrease with age; 🡽 increase with age; ns, no significant differences between groups but correlation with age; - , not included in the study | | | | | |
