# Supplementary material for: Age-Related Differences in Resting-State EEG and Allocentric Spatial Working Memory Performance
Source: Front Aging Neurosci. 2021 Nov 4;13:704362. doi: 10.3389/fnagi.2021.704362 (PMC8600362; doi:10.3389/fnagi.2021.704362)
Supplement: Supplementary Material 2 — Studies on age-group changes in alpha activity: age ranges considered, EEG parameters extracted and main findings. [file Table_2.docx]

|  | |  | |  | |  | | |  | |  | |
| --- | --- | --- | --- | --- | --- | --- | --- | --- | --- | --- | --- | --- |
| **Supplementary Material 2.** Studies on age-related changes in alpha activity: age ranges considered, EEG parameters extracted and main findings. | | | | | | | | | | | |  |
| **Study** | | **Mean age and/or age range** | | **Frequency band** | | **Parameters extracted** | | | **Results eyes open** | | **Results eyes closed** |  |
| *Volf & Gluhih, 2011* | | 22 vs 65 | | 1: 8-10Hz / 2: 10-13Hz | | absolute power (μV^2^/Hz; log transformed) | | | **ns** | | 1: **ns** / 2: 🡾 |  |
|  | |  | |  | |  | | |  | |  |  |
| *Barry & De Blasio, 2017* | | 20 vs 68 | | 8-13Hz | | absolute amplitude (μV) | | | 🡾 | | 🡾 |  |
|  | |  | |  | |  | | |  | |  |  |
| *Vysata et al., 2012* | | 20-70 | | 8-12.5Hz | | absolute power (μV^2^/Hz)  relative power (μV^2^/Hz) | | | - | | 🡾, linear regression  🡾, linear regression |  |
|  | |  | |  | |  | | |  | |  |  |
| *Breslau et al., 1989* | | 23 vs 70 | | 8.2-12.9Hz | | absolute amplitude (μV) | | | - | | 🡾 |  |
|  | |  | |  | |  | | |  | |  |  |
| *Hartikainen et al., 1992* | | 31 vs 53 vs 71 | | 7.6-13.9Hz  IAPF | | absolute amplitude (μV)  absolute power (μV^2^)  relative amplitude and power  peak frequency left occipital | | | -  -  -  - | | **ns**  **ns**  **ns**  **ns** |  |
|  | |  | |  | |  | | |  | |  |  |
| *Reichert et al., 2016* | | 25 vs 48 vs 68 | | 1: 8-10Hz / 2: 10-12Hz | | relative power (μV^2^; log transformed)  peak frequency at Pz | | | **ns**  **ns** | | **1: ns / 2:** 🡾, 25 ≅ 48 > 68  **ns** |  |
|  | |  | |  | |  | | |  | |  |  |
| *Widagdo et al., 1998* | | 29 vs 73 | | 8-12.5Hz | | relative power (-) | | | **ns** | | 🡾 |  |
|  | |  | |  | |  | | |  | |  |  |
| *Trammell et al., 2017* | | 21 vs 73 | | 8-12Hz | | relative power (-)  IAPF occipital and parietal sites | | | -  - | | **ns,** correlation with age  🡾 |  |
|  | |  | |  | |  | | |  | |  |  |
| *Penttila et al., 1985* | | 33 vs 73 | | 7.57-13.92Hz | | relative power (-) | | | - | | **ns** |  |
|  | |  | | 4.15-13.92Hz | | Occipital peak frequency | | | - | | **ns** |  |
| *Ponomareva et al., 2017* | | 36 vs 62 | | 8-12.99Hz | | relative power (log transformed) | | | - | | **ns** |  |
|  | |  | |  | |  | | |  | |  |  |
| *Gaal et al., 2010* | | 22 vs 67 | | 1: 8-10Hz / 2: 10-12Hz | | absolute power (μV^2^/Hz) | | | **ns** | | **ns** |  |
|  | |  | | 8-12Hz | | Peak frequency | | | **ns** | | 🡾 |  |
| *Kononen & Partanen, 1993* | | 23-80 | | 7.57-13.92Hz | | absolute amplitude (μV; log values) | | | 🡽, correlation 23-80yrs (posterior)  **ns**, correlation 20-60yrs  🡽, correlation 60-80yrs (posterior) | | **ns** |  |
| *Scally et al., 2018* | | 20 vs 70 | | IAPF  upper α: 10-12Hz | | absolute power (log trans)  peak Frequency (Hz) all channels  absolute power (log trans) | | | -  -  - | | **ns**  🡾  🡾 |  |
|  | |  | |  | |  | | |  | |  |  |
| *Lodder & van Putten, 2011* | | 0-96 | | IAPF | | peak frequency (Hz) occipital | | | - | | 🡾 after age 20 (regression) |  |
|  | |  | |  | |  | | |  | |  |  |
| *Chiang et al., 2011* | | 6-86 | | IAPF 4-14Hz automated | | peak frequency (HZ) all channels  peak power (μV^2^/Hz^-1^) | | | -  - | | 🡾, after age 20  🡾 |  |
|  | |  | |  | |  | | |  | |  |  |
| *Knyazeva et al., 2018* | | 20-81 | | IAPF* 5-15Hz automated | | averaged peak frequency | | | - | | 🡾 regression analysis |  |
|  | |  | |  | |  | | |  | |  |  |
| *Fan et al., 2014* | | 35 vs 74 | | 8-12Hz | | absolute power (-) | | | - | | 🡾 |  |
|  | |  | |  | |  | | |  | |  |  |
| *Oken & Kaye, 1992* | | 20-99 (<65 vs >65) | | 8.25-13Hz | | IAPF 5-15Hz  relative power (log transformed) | | | - | | 🡾  🡾 |  |
|  | |  | |  | |  | | |  | |  |  |
| *Duffy et al., 1984* | | 30-80 (males only) | | 7-14Hz | | peak frequency at occipital sites  peak amplitude (μV) at occipital sites | | | -  - | | **ns**, no correlation with age  🡾 weak neg. correlation |  |
| *Duffy et al., 1993* | | 30-80 (groups by decades) | | 7-13 or 4-13Hz  7-13Hz | | mean frequency at Oz  absolute and relative amplitude at O1,Oz,O2 | | | - | | **ns**, 7-13Hz 🡾 4-13Hz two-way ANOVA age effect oldest vs youngest groups only  **ns** |  |
| *Williamson et al., 1990* | | 65-81 | | 8-12Hz | | absolute power (-) | | | - | | 🡾, correlation (males only) |  |
|  | |  | |  | |  | | |  | |  |  |
| *Matousek et al., 1966* | | 17-64 | | 1: 7.5-9.5Hz  2: 9.5-12.5Hz | | absolute amplitude (μV/sec ; log transformed) | | | - | | 1: **ns**  2: 🡾, correlation |  |
|  | |  | |  | |  |  | | | |  |  |
| *Giaquinto and Nolfe, 1986* | | 49 vs 71 | | 8-12.25Hz | | relative power (-)  mean frequency | | | -  - | | **ns**  **ns** |  |
|  | |  | |  | |  | | |  | |  |  |
| *Pollock et al., 1990* | | 56-76 | | 8.2-11.7Hz | | absolute amplitude (μV ; log transformed) | | | **ns**, correlation with age | | **ns**, no correlation with age |  |
|  | |  | |  | |  | | |  | |  |  |
| **Abbreviations and symbols**  Parameters extracted: units reported in each study provided in parenthesis; (-) units not described in the study.  Results: 🡾 decrease with age; 🡽 increase with age; ns, no significant differences between groups but correlation with age; - , not included in the study | | | | | | | | | | | |  |
