# Supplementary material for: Age-Related Differences in Resting-State EEG and Allocentric Spatial Working Memory Performance
Source: Front Aging Neurosci. 2021 Nov 4;13:704362. doi: 10.3389/fnagi.2021.704362 (PMC8600362; doi:10.3389/fnagi.2021.704362)
Supplement: Supplementary Material 3 — Studies on age-group changes in beta activity: age ranges considered, EEG parameters extracted and main findings. [file Table_3.docx]

| **Supplementary Material 3.** Studies on age-related changes in beta activity: age ranges considered, EEG parameters extracted and main findings. | | | | | |
| --- | --- | --- | --- | --- | --- |
| **Study** | **Mean age and/or age range** | **Frequency band** | **Parameters extracted** | **Results eyes open** | **Results eyes closed** |
| *Barry & De Blasio, 2017* | 20 vs 68 | 13.5-24Hz | absolute amplitude (μV) | 🡽 | 🡽 |
|  |  |  |  |  |  |
| *Volf & Gluhih, 2011* | 22 vs 65 | 1: 13-20Hz / 2: 20-30Hz | absolute power (μV^2^/Hz; log transformed) | 1: 🡽 / 2: 🡽 | 1: **ns** / 2: **ns** |
|  |  |  |  |  |  |
| *Vysata et al., 2012* | 20-70 | 13-29.5Hz | absolute power (μV^2^/Hz)  relative power (μV^2^/Hz) | -  - | 🡾, linear regression  🡽, linear regression |
|  |  |  |  |  |  |
| *Breslau et al., 1989* | 23 vs 70 | 13.3-19.9Hz | absolute amplitude (μV) | - | 🡾 |
| *Hartikainen et al., 1992* | 31 vs 53 vs 71 | 14.2-20Hz | absolute amplitude (μV)  absolute power (μV^2^)  relative amplitude and power | -  -  - | **ns**  **ns**  **ns** |
| *Widagdo et al., 1998* | 29 vs 73 | 1: 13-19.5 / 2: 20-26.5 / 3: 27-33.5Hz | relative power (-) | 🡽 | 🡽 |
|  |  |  |  |  |  |
| *Ponomareva et al., 2017* | 36 vs 62 | 1: 13-19.99 / 2: 20-30Hz | relative power (log transformed) | - | 🡽 |
|  |  |  |  |  |  |
| *Gaal et al., 2010* | 22 vs 67 | 1: 12-25Hz / 2: 25-40Hz | absolute power (μV^2^/Hz) | **ns** | **ns** |
|  |  |  |  |  |  |
| *Kononen & Partanen, 1993* | 23-80 | 14.16-20.02Hz | absolute amplitude (μV, log values) | 🡽, correlation 23-80yrs  🡽, correlation 20-60yrs  **ns**, correlation 60-80yrs | 🡽, correlation 23-80yrs  🡽, correlation 20-60yrs  🡽, correlation 60-80yrs |
|  |  |  |  |  |  |
| *Fan et al., 2014* | 35 vs 74 | 12-30Hz | absolute power (-) | - | 🡽 |
|  |  |  |  |  |  |
| *Oken & Kaye, 1992* | 20-99 | 1:13.25-20Hz | relative power (log transformed) | - | 1: **ns**, correlation |
| *Duffy et al., 1984* | 30-80 (males only) | 1: 12-15.75Hz  2: 16-19.75Hz  3: 20-23.75Hz  4: 24-27.75Hz  5: 28-31.75Hz | absolute amplitude (-)  relative amplitude | 🡽 2,3, correlation with age  🡽 2,3, correlation with age | -  - |
|  |  |  |  |  |  |
| *Duffy et al., 1993* | 30-80 (groups by decades) | 1: 12-15.5Hz  2: 16-19.5Hz  3: 20-23.5Hz  4: 24-27-5Hz | relative amplitude (log transformed) | 🡽 2,3, two-way ANOVA age effect | - |
|  |  |  |  |  |  |
| *Williamson et al., 1990* | 65-81 | 1: 12-18Hz  2: 18-26Hz | absolute power (-) | - | 🡾, correlation  🡾, correlation (only females) |
|  |  |  |  |  |  |
| *Giaquinto and Nolfe, 1986* | 49 vs 71 | 1: 12.5-20Hz  2: 20.25-32Hz | relative power (-) | - | **ns,** 1,2 |
|  |  |  |  |  |  |
| *Matousek et al., 1967* | 17-64 | 1: 12.5-17.5Hz  2: 17.5-25Hz | absolute amplitude (μV/sec, log transformed) | - | **ns**, 1,2 |
|  |  |  |  |  |  |
| *Pollock et al., 1990* | 56-76 | 1: 12.1-16Hz  2: 16.4-30Hz | absolute amplitude (μV, log transformed) | **ns**, correlation with age | **ns**, correlation with age |
|  |  |  |  |  |  |
| **Abbreviations and symbols**  Parameters extracted: units reported in each study provided in parenthesis; (-) units not described in the study.  Results: 🡾 decrease with age; 🡽 increase with age; ns, no significant differences between groups but correlation with age; - , not included in the study | | | | | |
