# Supplementary material for: Age-Related Differences in Resting-State EEG and Allocentric Spatial Working Memory Performance
Source: Front Aging Neurosci. 2021 Nov 4;13:704362. doi: 10.3389/fnagi.2021.704362 (PMC8600362; doi:10.3389/fnagi.2021.704362)
Supplement: Supplementary Material 4 — Studies on age-group changes in gamma activity: age ranges considered, EEG parameters extracted and main findings. [file Table_4.docx]

| **Supplementary Material 4.** Studies on age-related changes in gamma activity: age ranges considered, EEG parameters extracted and main findings. | | | | | |
| --- | --- | --- | --- | --- | --- |
| **Study** | **Mean age and/or age range** | **Frequency band** | **Parameters extracted** | **Results eyes open** | **Results eyes closed** |
| *Volf & Gluhih, 2011* | 22 vs 65 | 1: 30-40Hz / 2: 40-50Hz | absolute power (μV^2^/Hz; log transformed) | 1: 🡽 / 2: 🡽 | 1: **ns** / 2: **ns** |
|  |  |  |  |  |  |
| *Vysata et al., 2012* | 20-70 | 30-60Hz | absolute power (μV^2^/Hz)  relative power (μV^2^/Hz) | -  - | 🡾, linear regression  🡽, linear regression |
|  |  |  |  |  |  |
| *Fan et al., 2014* | 35 vs 74 | 30-100Hz | absolute power (-) | - | 🡽 |
|  |  |  |  |  |  |
| **Abbreviations and symbols**  Parameters extracted: units reported in each study provided in parenthesis; (-) units not described in the study.  Results: 🡾 decrease with age; 🡽 increase with age; ns, no significant differences between groups but correlation with age; - , not included in the study | | | | | |
